# Using machine learning to uncover the relation between age and life satisfaction

**DOI:** 10.1038/s41598-022-09018-x

**Published:** 2022-03-28

**Authors:** Micha Kaiser, Steffen Otterbach, Alfonso Sousa-Poza

**Affiliations:** 1 Department of Management, Society, and Communication, Copenhagen Business School, Dalgas Have 15, 2000 Frederiksberg, Denmark; 2 Institute for Health Care and Public Management, University of Hohenheim, Fruwirthstrasse 48, 70599 Stuttgart, Germany; 3 IZA, Bonn, Germany

**Keywords:** Human behaviour, Scientific data, Statistics

## Abstract

This study applies a machine learning (ML) approach to around 400,000 observations from the German Socio-Economic Panel to assess the relation between life satisfaction and age. We show that with our ML-based approach it is possible to isolate the effect of age on life satisfaction across the lifecycle without explicitly parameterizing the complex relationship between age and other covariates—this complex relation is taken into account by a feedforward neural network. Our results show a clear U-shape relation between age and life satisfaction across the lifespan, with a minimum at around 50 years of age.

## Introduction

There is much popular belief in the existence of a mid-life crisis, that is the notion that a period of unhappiness exists around the age of 40. A large body of literature dating back many decades addresses this issue, yet much controversy still remains^1–4^. In the past decade, this research topic has been invigorated by the publication of Blanchflower and Oswald^5^ which documents a U-shaped relation between age and life satisfaction in a cross-sectional sample of over 500,000 individuals in the United States and Europe, with the minimum in life satisfaction being reached between 36 and 53 years of age. Numerous studies followed and comprehensive literature reviews were conducted by López Ulloa et al.^6^ and Galambos et al.^7^, with the former covering 20 studies published before 2013 and the latter covering 29 studies published between 2014 and 2019. López Ulloa et al.^6^ conclude that "despite the numerous recent papers published on this topic, controversy regarding the effect that ageing has on life satisfaction still exists" (p. 241) and "in general, it is difficult to say with certainty whether the relationship between age and wellbeing across the lifespan is linear or convex" (p. 240). Galambos et al.^7^ go further by concluding that "given the body of evidence over recent years, we cannot conclude that there is a universal U shape in happiness" (p. 908) and "we believe the conclusion that happiness declines from late adolescence to midlife (the first half of the U shape) is premature, and possibly wrong" (p. 900). Unsurprisingly, this conclusion is not shared by Danny Blanchflower who recently conducted a large-scale analysis that identified a U-shape in age in one hundred and forty-five advanced and developing countries^8^. His conclusion: "No ifs, no buts, wellbeing is U-shaped in age" (p. 618). Blanchflower and Graham^9^ review the psychology literature and show that two of the studies cited by psychologists suggesting there are no U-shapes are in error. Their conclusion: "It remains puzzling then why many psychologists continue to suggest that wellbeing is unrelated to age" (p. 15).

Although much of the evidence points to a U-shape, one cannot deny that conflicting evidence exists. Depending on the data used, the definition of wellbeing, estimation technique, and choice of covariates, several different forms can be observed in the literature. The ideal basis for any analysis of wellbeing across the lifespan would obviously be panel data that follow representative individuals for this entire period^10^, yet such data are seldom available. Thus, the literature on this topic is primarily based on cross-sectional data or on panel data in which the average duration in the panel is relatively short. Although it is intriguing that very many cross-sectional studies around the world produce a U-shape, they are inadequate for drawing conclusions about within-person change in wellbeing across the life span^7,11^. In this regard, panel data is more useful, yet selectivity issues and different answering styles of panel participants are pervasive^10,12^.

A further methodological issue is the choice of covariates. If the researcher's aim is to capture the pure (or ceteris-paribus) age affect on wellbeing, then covariates need to be included. However, there is not much consensus as to which covariates should be included. Economists tend to use a large number of covariates (often termed "the usual suspects"), including income, gender, education, number of children, marriage, employment, non-participant, unemployed and health^10^. Blanchflower^8^ and Blanchflower and Graham^9^ show in their multinational studies that the U-shape holds, irrespective whether controls are used or not. However, some studies show that the use of controls matter^13,14^. Furthermore, many controls are not only dependent on age (e.g., income), but also on wellbeing itself (e.g., marriage). Thus, not only is the choice of covariates important, but the way in which they are modeled also matters.

Considering all these methodological challenges, it comes as no surprise that quite a wide spectrum of results and opinions exists. In this paper, we circumvent a number of these issues by applying a machine learning (ML) approach in which the choice of model is largely data driven^15^. Because the age−life satisfaction relation is complex and dependent on multiple factors, the ML algorithm's ability to fit complex and highly flexible functional forms to the data without overfitting^16,17^ makes it a particularly suitable analytic tool. In this paper we develop an approach based on a neural network which is able to account for complex interdependencies of covariates (including interactions with age), and also able to isolate a pure age effect.

## Data and methodology

### Dataset

The data is taken from the German Socio-Economic Panel (SOEP), sampled from around 20,000 to 30,000 individuals between 1992 and 2016. Our outcome of interest is life satisfaction, measured on a scale from 0 to 10 and modeled as a function of around 30 variables (depending on the type of model) that capture both socioeconomic and sociodemographic characteristics (see Table 1). In particular, we include gender (i.e., being female, 'yes' or 'no'), marital status ('married' or 'not married'), number of children, years of education, real income, and employment status (being employed, being unemployed, not being in the labour force)—features which are denoted "usual suspects" according to Frijters and Beatton^10^. In addition, we include the degree of disability and self-rated health status, the latter measured on a five-point scale ('poor', 'suboptimal', 'satisfactory', 'good', 'excellent') that is then recoded into a binary health dummy with the last two categories denoting good health. Finally, we include whether the survey was administered by an interviewer ('yes' or 'no'), whether a respondent is a homeowner ('yes' or 'no') and whether a person in need of care lives in the household ('yes' or 'no'). After excluding observations with missing or implausible information and focusing exclusively on individuals between the ages of 20 and 70, our final dataset consists of 381,279 observations.

**Table 1:** Descriptive statistics: variables.

	Measure	Mean	SD	Median
# Children	Numeric	0.75	1.05	0.00
Years of education	Numeric	12.18	2.7	11.50
Life satisfaction	Numeric (0–10)	7.07	1.75	7.00
Degree of disability	Numeric (0–100)	5.45	17.97	0.00
Age (20–70)	Numeric	44.8	13.36	44.00
Unemployed	Factor (no/yes)	0.06	0.23	0.00
Not in labor force	Factor (no/yes)	0.2	0.4	0.00
Homeowner	Factor (no/yes)	0.43	0.5	0.00
Person in need of care living in HH	Factor (no/yes)	0.03	0.17	0.00
Married	Factor (no/yes)	0.7	0.46	1.00
Female	Factor (no/yes)	0.53	0.5	1.00
Data collected by interviewer	Factor (no/yes)	0.59	0.49	1.00
Good health	Factor (no/yes)	0.53	0.5	1.00
Household income in real values	Numeric	2696.05	2834.83	2303.52
Income satisfaction	Numeric (0–10)	6.33	2.29	7.00
5-year cohorts (1920–1995)	factor (1–16)	–	–	–
N		381,279		

Complex dependency relationships characterize these control variables, and many of these variables are dependent on age, since most life events, such as marriage, the birth of children, and the acquisition of homeownership, take place at a particular stage of life. For example, for most people who have an uninterrupted educational biography from the time they enter primary school until they complete their school or university education, the number of years of education in this life stage is a linear function of age, whereas it becomes time-invariant after the highest educational degree is reached. It is also important to note that, in a conventional employment biography, income is also likely to change over the course of working life and is thus also a function of age. Furthermore, these variables are also interdependent in complex ways. For example, the fact that someone is married, has three children, and owns a home in which a person in need of care lives will affect labor market status and thus household income.

These complex interdependencies could be included in a conventional regression model only when using further strong assumptions. For example, whether and to what extent marriage affects life satisfaction to a different extent if married at a young age compared to later could be represented in a regression model by an interaction effect (married × age), in this case, assuming a linear relationship. If there is a non-linear relationship, the regression modeling (e.g., via higher-order polynomials of age) becomes very comprehensive and there is a risk that the model will suffer from multi-collinearity and over-specification. Neural networks are designed to take these complex interdependencies into account and map them comprehensively.

### Empirical strategy

To identify the life satisfaction pattern over the lifecycle, we need to define the real-valued functions *f,* and *g,* that map the random variables ${x}_{1}, {x}_{2},\ldots ,{x}_{n}$ (the features mentioned in the previous section, i.e., the potential predictors of life satisfaction) to the possible values of life satisfaction. Additionally, we assume that *h* is a real-valued function consisting of *a* (the individual's age, i.e., the domain of *a* is the set of ${\mathbb{R}}_{\ge 0}$). Given the following proposition, we claim that it is possible to isolate the effect of age on life satisfaction (proof in the “Appendix”):

#### Proposition

*If we define* $y {:=} f\left( {x_{1} ,x_{2} , \ldots ,x_{n} } \right) + h\left( a \right)$ *and *$z {:=} g\left({x}_{1},{x}_{2},\ldots ,{x}_{n}\right)$* to be given by functions of the random variables *${x}_{1},{x}_{2},\ldots ,{x}_{n}$* and a, and if we further assume that *$E[f\left({x}_{1},{x}_{2},\ldots ,{x}_{n}\right)-g\left({x}_{1},{x}_{2},\ldots ,{x}_{n}\right)|a]=0$* then h(a)* = $E[y-z|a]$*.*

The underlying idea is simple when viewed in a practical (or empirical) way: we think of *y* and *z* as predictions from two distinct supervised machine learning models, with the first model containing age as an additional predictor. We further assume that the average effect of all other factors on life satisfaction (apart from age) is equal across the two models, conditional on age (this implies that—on average—*f* and *g* show the same mapping dependent on age). In that case, the lifecycle pattern of life satisfaction will result from the average differences of the predictions. The main advantage here is obvious: assuming that the assumptions hold, and assuming that we can (empirically) identify *f* and *g*, our method implies that we can identify the shape of life satisfaction across the lifecycle without parameterizing it a priori (as is common in this literatrure). Moreover, suppose life satisfaction is continuous in age and follows a U-shape. In that case, its empirical counterpart *h* (i.e. the average differences in predictions between the two ML models) should have precisely two roots according to Bolzano's theorem, or more generally, the resulting graph of *h* should mimic a U-shaped pattern.

### Feedforward neural network

In order to predict *y* (and *z*) we first split our data randomly into a training set (containing 80% of the observations) and a test set (containing 20% of the observations). Based on the previous considerations, we then train a feedforward neural network model that includes all features—including age (and cohorts)—on the training data and evaluate its performance on the test set (we call this the baseline model in the following). We assume that this baseline model gives us a valid prediction of $y$*.*

Since the model's performance is highly sensitive to the hyperparameters used, we have to check many possible combinations of all potential parameters and select a combination that shows a low error on both the training and the test data. In particular, we tune the following parameters to obtain the best predictive result by avoiding over-or underfitting: the *number of epochs* and the *batch size* used during the training*;* the *number of nodes per layer;* the *number of layers* and the *dropout rate*; *activation functions* used*,* the *optimization algorithm;* and the *learning rate.*

The final specification, that is the one that gives the lowest mean square error (MSE) for the training and test data, is described in Table 2. Thus, our baseline model consists of 3 layers (one input layer and two hidden layers), with 31 input nodes and 62 hidden nodes evenly distributed among the two hidden layers. It uses Leaky-ReLU activation functions in the hidden layers and ReLU activation for the final output layer (note that we choose ReLU instead of Leaky-ReLU activation to avoid negative values of *y*. See^18^, for a detailed comparison of ReLU and Leaky-ReLU). The dropout rate is set to 0, which is probably related to the fact that the model consists of "only" 2 hidden layers and is therefore not as susceptible to overfitting compared to "deeper" model architectures (see^19^ for a discussion of the use and benefit of dropout rates in deep neural networks).

**Table 2:** Model characteristics.

**Basic characteristics**				
Type of neural net	Feedforward neural network			
Loss function	MSE			
**Training characteristics**				
Batch size	1			
Number of epochs	20			
Optimization	Adam optimization			
Learning rate	0.0001			
Initial weight distribution	${w}_{i}^{l}\sim N(0, \frac{1}{\sqrt{31}})$			
**Layer characteristics**				

Layer	Input (1)	Hidden (2)	Hidden (3)	Output (4)
Features/nodes	31 (Including 16 cohort dummies and age)	31	31	1
Activation	Input	Leaky-ReLU	Leaky-ReLU	ReLU
Dropout rate	0	0	0	0
**Performance**				
Training error	*1.80*			
Test error	*1.95*			

The actual optimization is performed during 20 epochs (with a batch size of 1 per iteration) using an Adam optimizer with a learning rate of 0.0001 (again, note that we test different optimization techniques and learning rates. For a detailed discussion of the Adam algorithm, see^20^. Finally, before the first iteration of the training process, we initialize the weights according to a normal distribution with a mean of 0 and a standard deviation of $\frac{1}{\sqrt{31}}$ (i.e., ${w}_{i}^{l}\sim N(0, \frac{1}{\sqrt{31}})$ for the *i-*th weight in the *l*th layer).

Starting from this baseline model, we next train an additional model with exactly the same specifications (i.e., the same hyperparameter values), excluding age (since the only missing predictor is age, we argue that this model's predictions reflect *z*)*.* To rule out that cohort rather than age effects drive the difference between *y* and *z*, we also train two other models—one without cohort information and one without age as well as cohort information.

All these steps provide us with what we need to finally compute *h(a)* (i.e., the shape of life satisfaction over the life cycle), which is done by taking the difference of the predictions on the test data of the baseline model and the models without age, cohorts, and age and cohorts, respectively (the full code is available at https://github.com/13kaiser/life_satisfaction/).

### Random forest and partial least square regression

To check the robustness of our empirical strategy, we also apply a random forest (RF) model and a partial least squares (PLS) regression. While we perform a grid search over the *maximum number of trees* and the *maximum depth* of each tree when training the RF, we only tune the *maximum number of components* when training the PLS model. After training and grid search, our final baseline RF model consists of 200 trees, each with a maximum depth of 15, while the final PLS model consists of 13 components (the full code is available at https://github.com/13kaiser/life_satisfaction/).

## Results

**Figure 1:**
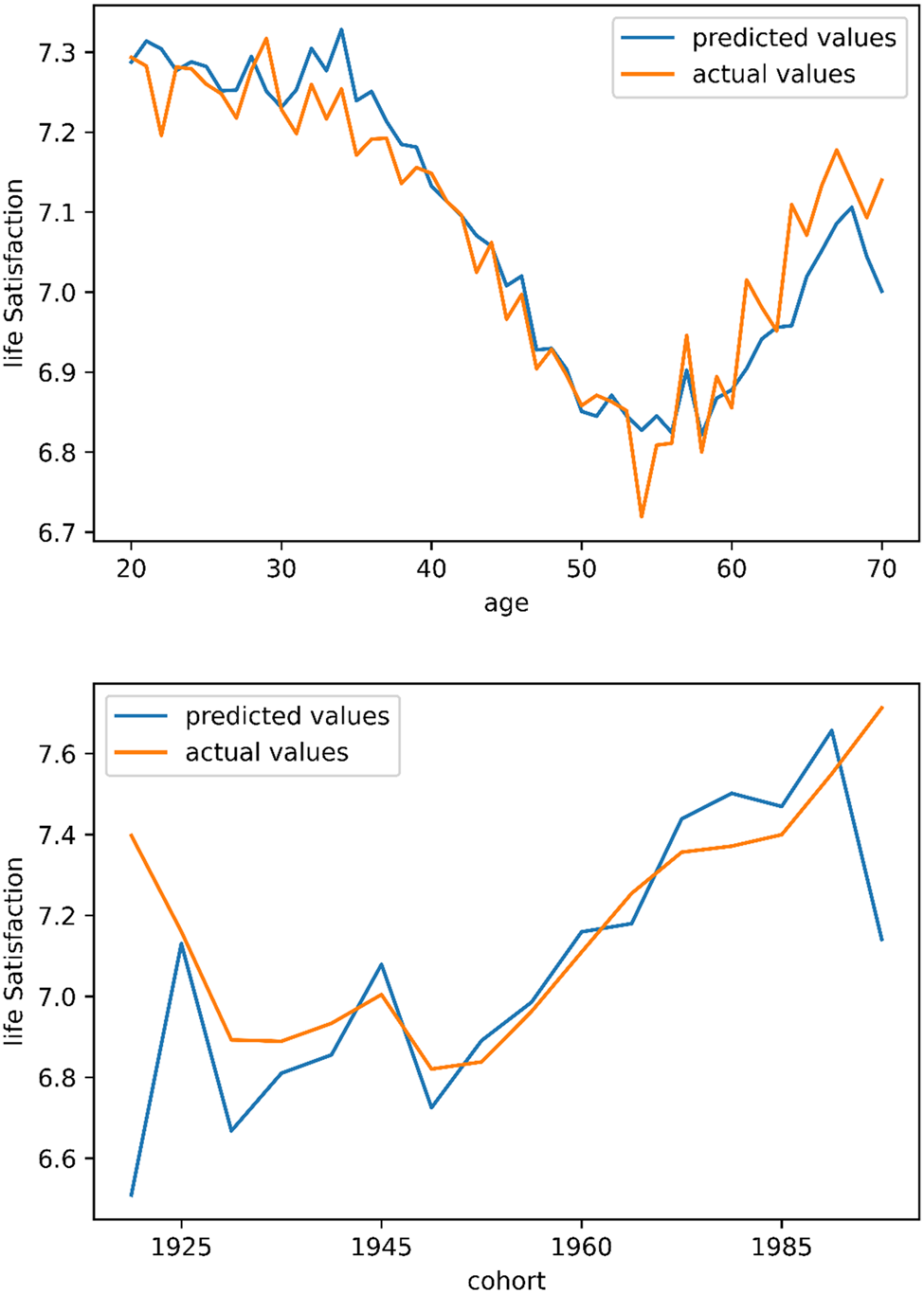
Predictions of life satisfaction on test data. Neural network predictions of life satisfaction as a function of age and cohorts. The blue line indicates the predicted values given the test data, while the orange line shows the actual values within the test data.

**Figure 2:**
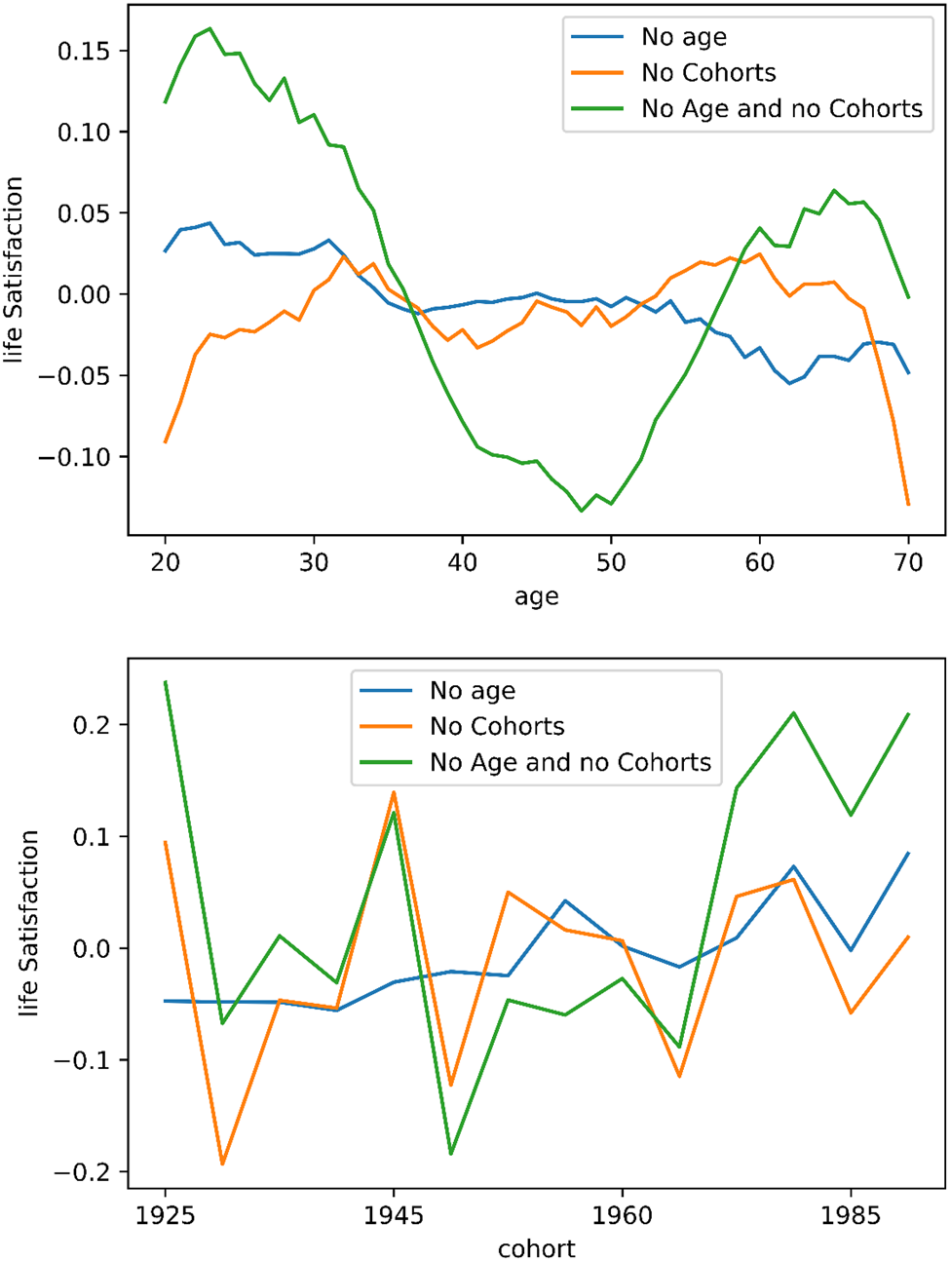
Predictions of life satisfaction on test data. The figure shows the differences in the baseline model predictions (including age and cohorts) and the models excluding age, cohort, and age and cohort. Differences are shown by age (top panel) and cohorts (bottom panel).

The resulting baseline model shows a training MSE of 1.80 and a test error of around 1.95. As can be seen in Fig. 1, the predicted values correspond very closely to the actual values. This is in itself an interesting result as it shows that a relatively limited number of features that capture demographics, economic situation, and health status, combined with a neural network, can predict life satisfaction very accurately. The bottom panel of Fig. 1 depicts the precited and actual values for different cohorts. These precited and actual values also correspond quite closely to each other, with the exception of the very young and very old cohorts for which there are limited observations. The top panel of Fig. 2 plots three versions of the *h(a)* function using the test data. The green function depicts the difference between the predictions made in the baseline model (*y values*) and the same model without including age or cohort variables (*z values*). Interestingly, a clear U-shape emerges with positive differences up to the age of around 40, negative differences between the ages of 40 and 60, and positive differences thereafter. The largest negative difference is found at around the age of 50. This function thus reveals that when omitting age and cohort variables in the neural network, life satisfaction levels will be underestimated among the young and the old, and overestimated among middle-aged individuals. In the top panel of Fig. 2, the blue line shows the difference when including cohorts but excluding age in the calculation of the z values. The orange line shows the difference when including age variables but excluding cohorts in the calculation of the z values. In both cases, we observe a relatively flat function around the value of zero. In other words, if we include age but omit cohorts, or if we include cohorts but omit age, the U-shape vanishes. This result is not very surprising as cohorts are defined using the age variable—and clearly, the neural network observes age, proxied by cohorts, when using the cohort variables. In the lower panel of Fig. 2 we observe the *h(a)* function plotted for different cohorts. If we focus on the green line that does not include any age or cohort variables, then we note that, in general, cohorts born immediately after the second world war (1950–1965) have *h(a)* values below zero, implying that when omitting age and cohort variables in the neural network, life satisfaction levels will be underestimated among these cohorts. Furthermore, the graph indicates that an overestimation will take place among cohorts born after 1965.

Concerning the RF and the PLS, we find that both additional models confirm the results of our main specification. That is, once we exclude age and cohorts, the U-shaped pattern emerges. Interestingly, the RF model seems to be more sensitive to the exclusion of age than to the exclusion of cohorts (no U-shape emerges when we exclude only cohorts), while the opposite is true for the PLS regression (see Figures S1 and S2 in the “Appendix”).

## Discussion and conclusions

Specifying life satisfaction models that can determine the pure age effect on life satisfaction is challenging and the topic of much controversy. Not only does life satisfaction depend on numerous variables, but many of these variables are also, in turn, dependent on age. The relations between these variables are also complex. ML techniques such as neural networks offer a valuable tool for modeling any complex relations in large, rich data sets, but particularly those between age and life satisfaction, a multidimensional construct that is intricately intertwined with myriad other factors. Not only is a data-driven technique the best choice for such complicated modeling, but it is also the most feasible approach to handling the approximately 400,000 observations in the SOEP and their relations to a multiplicity of variables.

It is well-known that there are complex interdependencies between age and various covariates (such as marriage, age, and income), yet these relationships are not a priori known and therefore cannot be meaningfully modeled parametrically. The existing literature on the age-life satisfaction relation does not try to model such interdependencies but usually merely adds age as one independent variable. In our approach, we actually solve the problem of interdependencies among the different determinants of life satisfaction. The underlying assumption in our analysis which allows us to isolate the age effect, is that these dependencies do not play a crucial role within a given age or birth cohort, but they do matter across ages and cohorts (see Proposition). With this assumption, we can isolate a possible age effect across age and birth cohorts.

Our assumption that these dependencies do not play a crucial role *within* a given age or birth cohort implies that our model should be interpreted as an estimate of the long-run effect of age on life satisfaction, which admittedly ignores possible short-run age effects. However, we do not believe that short-term age interdependencies are the key determinants of the long-term relation between age and life satisfaction, because, by the very nature of our model, these are interdependencies of age and other covariates that take place within a year (or 5 years in the case of birth cohorts).

Using this intuitive approach at isolating the age effect with ML techniques, our results reveal that, when omitting age variables in a neural network that predicts life satisfaction, life satisfaction levels will be underestimated among the young and the old overestimated among middle-aged individuals. This indicates that life satisfaction is U-shaped across the lifespan, with a minimum at around 50 years of age. With the existence of large panel data sets in many countries, it would be interesting for future research to determine the generality of the U-shape relation between age and life satisfaction using such ML techniques. Although the focus of this paper was on the widely discussed age-life satisfaction relation, the approach taken in this paper could also be used to assess how other factors such as income and education affect well-being across the lifespan.

## Supplementary Information

Supplementary Information.

## Data Availability

The data used in this publication were provided to us by the Socio-Economic Panel (SOEP) at the German Institute for Economic Research (DIW), Berlin. The data are publicly available after registration at www.diw.de/en/diw_02.c.242211.en/criteria_fdz_soep.html. Because the SOEP data are fully anonymized secondary data, ethical approval was not required.

## References

[1] Brim OG (1992). Ambition.

[2] Chiriboga DA, Lachman ME, James JB (1997). Crisis, challenge, and stability in the middle years. Multiple Paths of Midlife Development.

[3] McCrae R, Costa P (1990). Personality in Adulthood.

[4] Wethington E (2000). Expecting stress: Americans and the “midlife crisis”. Motiv. Emot..

[5] Blanchflower DG, Oswald AJ (2008). Is wellbeing U-shaped over the life cycle?. Soc. Sci. Med..

[6] López Ulloa BF, Møller V, Sousa-Poza A (2013). How does subjective wellbeing evolve with age? A literature review. J. Popul. Ageing.

[7] Galambos NL, Krahn HJ, Johnson MD, Lachman ME (2020). The U shape of happiness across the life course: Expanding the discussion. Perspect. Psychol. Sci..

[8] Blanchflower DG (2021). Is happiness U-shaped everywhere? Age and subjective wellbeing in 145 countries. J. Popul. Econ..

[9] Blanchflower, D. G., & Graham, C. L. (2020). The Mid-Life Dip in Well-Being: Economists (Who Find It) Versus Psychologists (Who Don't)! (No. w26888). National Bureau of Economic Research.

[10] Frijters P, Beatton T (2012). The mystery of the U-shaped relationship between happiness an age. J. Econ. Behav. Organ..

[11] Li N (2016). Multidimensionality of longitudinal data: Unlocking the age-happiness puzzle. Soc. Indic. Res..

[12] Kassenboehmer SC, Haisken-DeNew JP (2012). Heresy or enlightment? The wellbeing age U-shape effect is flat. Econ. Lett..

[13] Hellevik O (2017). The U-shaped age–happiness relationship: Real or methodological artifact?. Qual. Quant..

[14] Helliwell, J. F., Huang, H., Norton, M. B., & Wang, S. Happiness at different ages: The social context matters. In *The economics of happiness*. 455–481 (Springer, 2019).

[15] Athey S, Agrawal AK, Gans J, Goldfarb A (2019). The impact of machine learning on economics. The Economics of Artificial Intelligence: An Agenda.

[16] Mullainathan S, Spiess J (2017). Machine learning: An applied econometric approach. J. Econ. Perspect..

[17] Varian H (2014). Big data: New tricks for econometrics. J. Econ. Perspect..

[18] Dubey, A. K., & Jain, V. Comparative study of convolution neural network's relu and leaky-relu activation functions. In *Applications of Computing, Automation and Wireless Systems in Electrical Engineering*. 873–880 (Springer, 2019).

[19] Hinton, G. E., Srivastava, N., Krizhevsky, A., Sutskever, I., & Salakhutdinov, R. R. Improving neural networks by preventing co-adaptation of feature detectors. arXiv:1207.0580 (2012).

[20] Kingma, D. P., & Ba, J. Adam: A method for stochastic optimization. arXiv:1412.6980 (2014).

