# Supplementary material for: Using machine learning to uncover the relation between age and life satisfaction
Source: Sci Rep. 2022 Mar 28;12:5263. doi: 10.1038/s41598-022-09018-x (PMC8960822; doi:10.1038/s41598-022-09018-x)
Supplement: Supplementary file 1 — Supplementary Information. [file 41598_2022_9018_MOESM1_ESM.docx]

**Proof of Proposition**

*Discrete case:* $E\left[ y-z | a \right]=E\left[ f\left( x_{1},x_{2},\ldots,x_{n} \right)+h\left( a \right) | a \right]- E\left[ g\left( x_{1},x_{2},\ldots,x_{n} \right) | a \right]$

$$=\sum_{x_{1}} \sum_{\ldots} \sum_{x_{n}} f\left( x_{1},x_{2},\ldots,x_{n} \right)p_{x_{1},x_{2},\ldots,x_{n}|a}(x_{1},x_{2},\ldots,x_{n}\left| a \right)-\sum_{x_{1}} \sum_{\ldots} \sum_{x_{n}} g\left( x_{1},x_{2},\ldots,x_{n} \right)p_{x_{1},x_{2},\ldots,x_{n}|a}(x_{1},x_{2},\ldots,x_{n}\left| a \right)+h\left( a \right)$$

$$= E\left[ f\left( x_{1},x_{2},\ldots,x_{n} \right)-g\left( x_{1},x_{2},\ldots,x_{n} \right) | a \right]+h\left( a \right)=h(a)$$

We can easily prove the proposition also for the continuous case if we assume that the conditional joint density function $\varphi: \mathbb{R}^{n}\mathbb{\to R}$ exists.

*Continuous case:* $E\left[ y-z | a \right]=E\left[ f\left( x_{1},x_{2},\ldots,x_{n} \right)+h\left( a \right) | a \right]- E\left[ g\left( x_{1},x_{2},\ldots,x_{n} \right) | a \right]$

$$=\iint\ldots\int f\left( x_{1},x_{2},\ldots,x_{n} \right)\varphi_{x_{1},x_{2},\ldots,x_{n}|a}(x_{1},x_{2},\ldots,x_{n}\left| a \right)dx_{1}dx_{2}\ldots dx_{n}-\iint\ldots\int g\left( x_{1},x_{2},\ldots,x_{n} \right)\varphi_{x_{1},x_{2},\ldots,x_{n}|a}(x_{1},x_{2},\ldots,x_{n}\left| a \right)dx_{1}dx_{2}\ldots dx_{n}+h(a)$$

$$=E\left[ f\left( x_{1},x_{2},\ldots,x_{n} \right)-g\left( x_{1},x_{2},\ldots,x_{n} \right) | a \right]+h\left( a \right)=h(a)$$

| 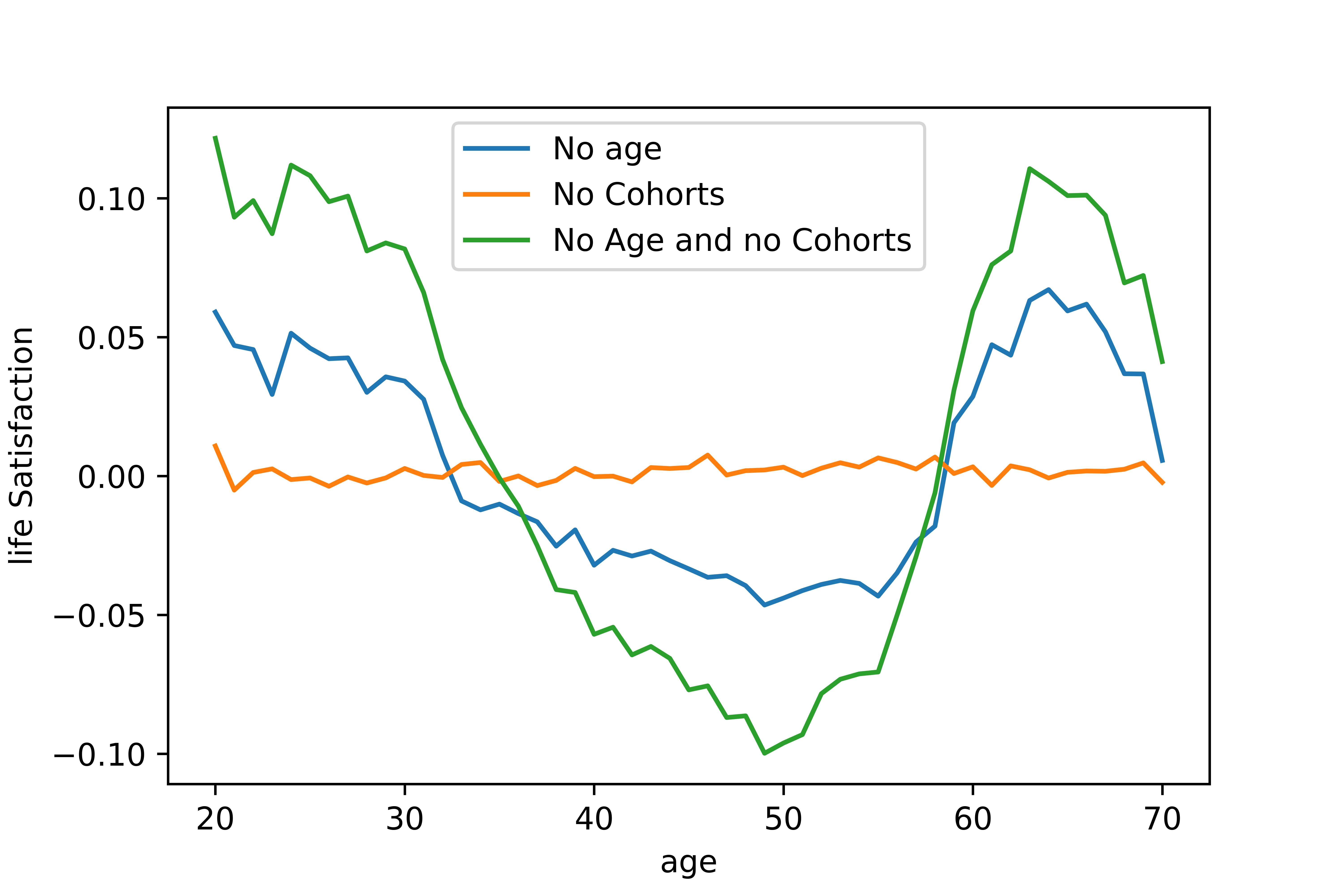 |
| --- |

*Figure S1: Predictions of life satisfaction on test data using a Random Forest
The figure shows the differences in the baseline model predictions (including age and cohorts) and the models excluding age, cohort, and age and cohort. The model consists of 200 trees, with each tree having a maximum depth of 15.*

| 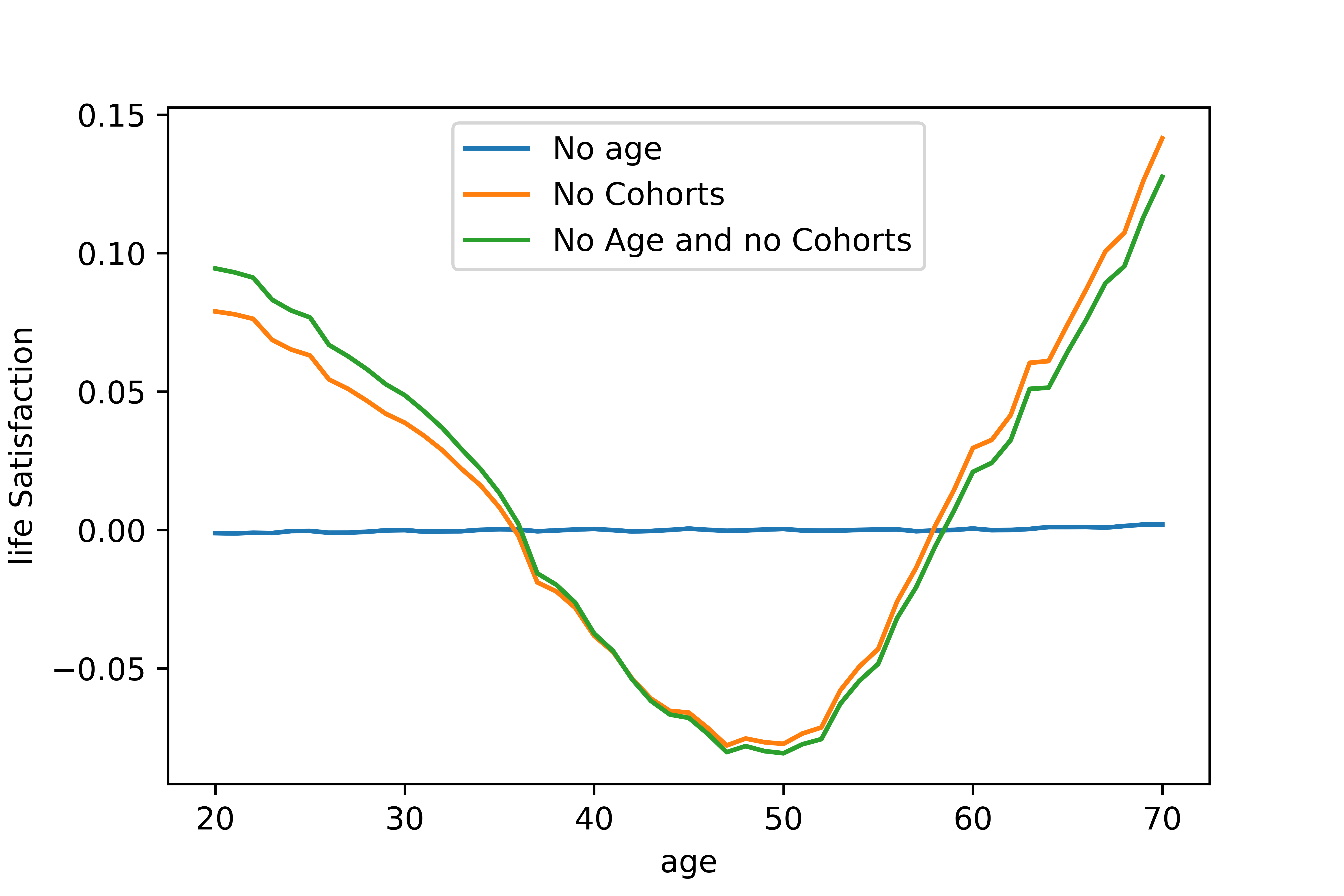 |
| --- |

*Figure S2: Predictions of life satisfaction on test data using a Partial Least Squares regression
The figure shows the differences in the baseline model predictions (including age and cohorts) and the models excluding age, cohort, and age and cohort. The Partial Least Squares model consists of 13 components.*
